# Drag Effect of Economic Growth and Its Spatial Differences under the Constraints of Resources and Environment: Empirical Findings from China’s Yellow River Basin

**DOI:** 10.3390/ijerph19053027

**Published:** 2022-03-04

**Authors:** Yujiao Zhou, Ding Li, Weifeng Li, Dong Mei, Jianyi Zhong

**Affiliations:** 1 School of Economics, Southwestern University of Finance and Economics, Chengdu 611130, China; 2 School of Public Administration, Southwestern University of Finance and Economics, Chengdu 611130, China; liding@vip.sina.com; 3 PBC School of Finance, Tsinghua University, Beijing 100084, China; liwf@pbcsf.tsinghua.edu.cn; 4 Institute of Agricultural Resources and Regional Planning (IARRP), Chinese Academy of Agricultural Sciences, Beijing 100081, China; 5 Institute of Finance and Economics, Guangzhou Huali Science and Technology Vocational College, Guangzhou 511325, China; zhongjyhuali@163.com

**Keywords:** resources constraints, environmental pollution, drag effect, spatial effect

## Abstract

As the global economic development intensifies the plunder of resources and the environment, the constraints are becoming more and more obvious. Based on the background of the strategy for ecological conservation and high-quality development of the Yellow River Basin, this paper intends to construct a resource-environment-constrained economic growth drag effect model and a spatial Dubin model, and explore the economic growth drag effect and its spatial differences in the Yellow River Basin under the constraints of resources and environment. The study found that the total drag effects of the overall economic growth of the Yellow River Basin that were obtained by the classic panel model without spatial effects is significantly negative. This is consistent with the conclusion that the average total drag effects of 80 prefecture-level cities is negative. The total drag effects of the overall economic growth of the Yellow River Basin changes from unconstrained to medium-constrained after adding spatial constraints, indicating that the spatial correlation of factors will restrict economic growth. From the level of the Yellow River sub-catchment, the total drag effect of the direct effects of the upper, middle, and lower reaches of the Yellow River is consistent with the total drag effect of the total effect. It shows that the upper economic growth is strongly constrained by the local resources and environment, while the downstream is strongly constrained by the adjacent resources and the environment. The research results provide references for resolving the resources and environment constraints in the Yellow River Basin. It provides useful inspiration for promoting ecological protection and high-quality development strategies in the Yellow River Basin.

## 1. Introduction

Resource and environmental issues have always been a major problem facing the global economic development, playing a role in either hindering or promoting economic development, and as economic development intensifies the plunder of resources and the environment, the constraints are becoming more and more obvious. In China, the Yellow River still provides irrigation for several provinces and hundreds of millions of people. How to make good use of river resources and protect the environment is a problem that the Chinese government is currently trying to solve. The government has put forward the goal of achieving new progress in the construction of ecological civilization, which includes improving the efficiency of energy resource allocation, continuously reducing the total discharge of major pollutants, and continuously improving the ecological environment. A win-win development between them is the basis for realizing the ecological environmental protection and a high-quality development strategy of the Yellow River Basin.

In recent years, with the economic development of the Yellow River Basin, the contradiction between resource finiteness and the infinite demand for resources due to economic growth, as well as the contradiction between environmental pollution and economic growth, have become more and more significant. At present, the Yellow River Basin is facing a decline in the use rate of energy resources, a relative shortage of water resources, prominent land desertification, a downward trend in the available land resources, and three industrial wastes. The problem can be attributed to the environmental damage and ecological fragility of the Yellow River Basin that has been caused by resource utilization and environmental pollution, which may drag or restrict social and economic development to a certain extent, that is, the drag effect of economic growth. Traditional economic theory believes that natural resources should be the driving force of economic development, that is, emphasizing the positive role of natural resources without considering its restraint on economic development. As the resource carrying capacity is limited, once it exceeds its threshold range, its effect may be reversed. At the same time, because the processes of economic development and natural resource utilization are time-dependent and spatially relevant, the economic development of natural resources restraint becomes non-linear and spatially-dependent [1]. Therefore, attention should be paid to the temporal and spatial constraints of natural resources on economic development. In addition, the environmental carrying capacity is also limited. Once the pollution that exceeds the environmental capacity continues to increase, economic development may be unsustainable, and environmental pollution has strong timelines and spatial relevance. This may have side effects on itself, the surrounding environment, and even economic development. At the same time, the regional economic development gap of the Yellow River Basin is becoming more and more obvious. This phenomenon has become a major problem facing the economic development of the Yellow River Basin [2]. 

The growth drag effect, which can also be called growth resistance or damping effect, generally refers to the degree of restriction of economic factors on economic growth, that is, the difference between the unconstrained and constrained economic growth rate. The qualitative and quantitative analysis of the growth drag effect in foreign countries is earlier and more in-depth, mainly focusing on: First, expand the economic growth drag effect model. Nordhus incorporated natural resources on the basis of the Solow model and constructed a neo-classical growth model to explore the drag effects of natural resources [3]. Barbier extended the Romer-Stiglitz model, adding endogenous technological progress and labor, to study the restraining effect of natural resources on endogenous economic growth [4]. Bruvoll used a dynamic CGE (Computable general equilibrium) model to measure the damping effect of environmental pollution on economic growth [5]. Of course, the most widely used is Romer that established the classic drag effect calculation model on resources and land constraints when defining the economic growth drag effect [6]. Later, scholars mainly started from this model to expand and deepen the model. Tsur et al. joined the learning by using model to analyze whether resource constraints can be eliminated in different situations in the process of economic growth [7]. Second, the expansion of research perspectives and research objects. After sorting and summarizing the relevant foreign literature, it was found that the theory of economic growth drag effect is mainly used to measure natural resources, land resources, environmental pollution, environmental policy, unemployment, power infrastructure, etc. [3,4,5,6,7,8,9,10,11,12]. Scholars have transitioned from the early research on the drag effect of economic factors to the policy drag effect (environmental policy, financial policy, fiscal policy, etc.). In addition, the drag effect of regional economic growth has been extended to the drag effect of various industries, and the application field of drag effect theory has been expanded. Third, diversifying the water mix can help reduce the drag effects of economic growth. Sangmin believed that diversifying the water infrastructure helps address water risks [13]. Boryczko analyzed how diversifying water supplies can help reduce the risk of mitigating water shortages [14]. Morton believed that the reuse of drinking water will help to diversify the water source mix and strengthen the management of water resources [15].

Domestically, beneficial explorations have also been carried out on the theory of growth drag effects. On the one hand, it is the expansion of research objects. The drag effect model measures more independent variables. Natural resources, energy resources, water resources, land resources, population resources, environmental pollution, marine resources, etc. are all included in the drag effect model for economic growth drag effect analysis. Xue et al., based on Romer’s classic drag effect calculation model, calculated the drag effects of China’s economic growth by analyzing natural resources and land resources [16]. Cui and Wang et al. analyzed the drag effects of land resources from overall and regional economic growth, respectively [17,18], Luo improved the original land resource drag effect model from the perspective of virtual land growth [19]. Xie et al., Liu et al., and Wan et al. analyzed the drag effect of economic growth from the perspective of water and land resource constraints [20,21,22]. The second is the expansion of research fields. On the one hand, Zhao et al. and Cao et al. subdivided the research scope into the Yangtze River Delta [23,24]. Sun et al. studied the restraining effect of water resources on economic growth in arid areas [25]. On the other hand, with the deepening of research, energy drag efficiency was included in drag efficiency research [26,27,28,29], and resources and environmental drag efficiency. It also gradually appeared in the research field of vision. Liu et al. and Li et al. took the urbanization process and the industrialization process as the perspectives to construct and analyze the resource and environmental drag effects from the variables of land, energy, water resources, and environmental pollution [30,31]. Gao et al. selected Shaanxi and national data for comparative analysis of the energy and environmental constraints in the process of urbanization [32]. Li et al. extended the classic drag effect model to measure the degree of energy and environmental constraints on economic growth in poverty-stricken areas, etc. [2].

Through the analysis of the domestic and foreign literature, the existing research results provide a certain theoretical basis and direction guidance for carrying out research on resources and environmental drag effects. There is still room for development and improvement, which is mainly reflected in: First, the existing resource and environmental drag effect model is based on the province or the country and has not studied the data at the prefecture-level city; this study may obtain more detailed results and discussions. Second, the existing environmental drag effects are mostly measured by CO_2_ or SO_2_ emissions. Environmental pollution should be a collection of multi-dimensional pollution variables [33], which can effectively enhance the interpretation of existing explanatory variables. Third, the existing resource and environmental drag effect models rarely involve spatial correlation and spatial dependence. The Yellow River Basin, whether it is water resources, energy resources, environmental pollution, and other adjacent areas, will interact with each other to a certain extent, that is, the economic growth of a region is not only affected by economic factors in the region but also by the neighboring regions. Based on this, this paper uses the 2003–2018 panel data of 80 prefecture-level cities in the Yellow River Basin, and adopts the assumption of constant returns to scale, using energy resources, water resources, land resources, industrial wastewater emissions, industrial SO_2_ emissions, and industrial smoke, (powder) dust emissions, etc., expands the Romer’s classic drag effect model to construct an economic growth model under the constraints of resources and environment in the Yellow River Basin, and analyze the drag-efficiency and spatial effects of economic growth in the Yellow River Basin through the classic panel model and the spatial Durbin model (SDM). In addition, from the prefecture-level cities and the Yellow River Basin, the regional spatial difference analysis is carried out to find the law of spatial differentiation and influencing factors.

## 2. Material and Methods

### 2.1. Variable Selection and Data Sources

#### 2.1.1. Research Object

**Figure 1:**
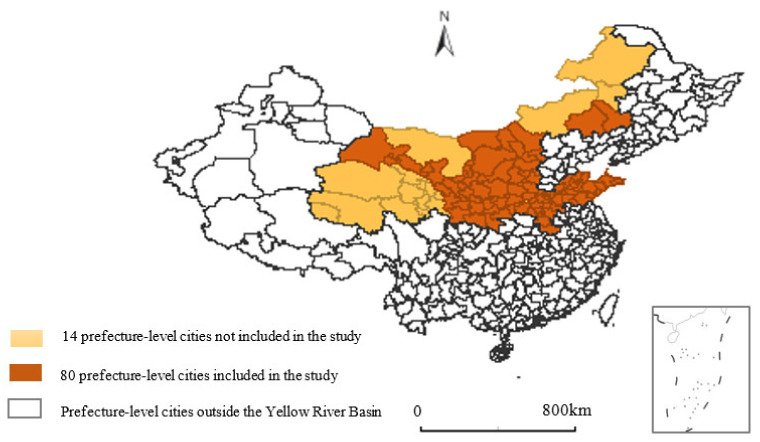
The research area of the Yellow River Basin.

It can be seen in Figure 1 that the Yellow River flows from west to east, with a large drainage area, flowing through 9 provinces including Qinghai, Sichuan, Gansu, Ningxia, Inner Mongolia, Shanxi, Shaanxi, Henan, and Shandong. This paper takes 80 prefecture-level cities in the Yellow River Basin as the research object (prefecture-level cities with serious data missing have been eliminated). Regarding the division of the upper, middle, and lower reaches of the Yellow River Basin, this paper refers to the practice of Sun et al. [34]. The upper reaches of the Yellow River include Qinghai, Gansu, and Ningxia; the middle reaches include Inner Mongolia, Shanxi, and Shaanxi; and the lower reaches include Henan and Shandong.

#### 2.1.2. Selection, Processing, and Data Sources of Specific Variables

(1) Local GDP (gross domestic product) (Y). This paper selected the regional GDP to measure the economic growth of cities in the Yellow River Basin. In order to eliminate the impact of price changes, the regional GDP was set at a constant price in 2003, and the actual regional GDP of each city was calculated based on the GDP index.

(2) Capital stock (K). This article selected the capital stock of each city from 2003 to 2018 and draws on Zhang’s inter-provincial capital stock method for calculation [35]. The specific calculation formula is as follows:

(1) $$K_{it}=K_{it-1}\left(1-\delta_{it}\right)+I_{it}$$

Among them, $K_{it}$ is the capital stock, and $K_{it-1}$ is the capital stock of the previous year; $I_{it}$ is the total fixed capital investment of the whole society; i represents the i-th prefecture-level city; t represents the t-th year; $\delta_{it}$ is the depreciation rate, which is 9.6%.

(3) Effective labor (AL). This article is based on Wang et al. [36]. human capital stock measurement standard to calculate human capital stock = average years of education * number of employees, of which the average years of education are 6 years of elementary school, 9 years of junior high school, 12 years of high school, and ordinary high school. This was calculated by the school for 16 years, and the number of employees was the number of employees in urban units.

(4) Resource indicators. Energy Resources (E). This paper also used the total gas supply (man-made, natural gas) as a measure of energy resources. Water resources (W) represented the total amount of water resources while land resources (R) were measured as the area of urban construction land.

(5) Environmental pollution indicators were used as measurement indicators for industrial wastewater discharge (B), Industrial SO_2_ discharge (S), and industrial smoke (dust) discharge (D).

#### 2.1.3. Data Sources and Descriptive Statistical Analysis

(1) Data source. The data came from the “China City Statistical Yearbook”, “China Urban and Rural Construction Statistical Yearbook”, “China Regional Statistical Yearbook”, “China Environment Statistical Yearbook”, 2004–2019 Provincial Statistical Yearbook and Prefecture-level City Accounting Yearbook, 2003–2018 Provincial Water Resources Bulletins, 2003–2018 National Economic and Social Development Statistical Communiqués of Prefectures and Cities, Provincial and City Portals, China Statistical Information Network, Qianzhan.com.

(2) Variable description. The descriptive statistics of the main variables are shown in Table 1.

### 2.2. Methods and Models

#### 2.2.1. Construction of a Drag Effect Model of Economic Growth in the Yellow River Basin under Resource and Environmental Constraints

Romer incorporated natural resources into the Solow economic growth model and constructed a classic drag effect calculation model that includes the dual constraints of land resources and natural resources to measure the restraining effects of natural resources and land resources on economic growth [6]. Through Cobb Douglas, the production function is simplified, and the drag effect model can be obtained:

(2) $$Y\left(t\right)=K{\left(t\right)}^{\alpha }R{\left(t\right)}^{\beta }T{\left(t\right)}^{\gamma }{\left[A\left(t\right)L\left(t\right)\right]}^{1-\alpha -\beta -\gamma }$$

Among them, $Y\left(t\right),\;K\left(t\right),\;R\left(t\right),\;T\left(t\right),\;A\left(t\right),\;L\left(t\right)$ are total output, capital stock, natural resources, land resources, technological progress, and labor input, respectively. The product of A(t), L(t) represents effective labor, α, β, γ represent the elasticity coefficients of capital stock, natural resources, and land resources, respectively, meeting the condition of α>0, β>0, γ>0, α+β+γ=1.

A study of the calculation of the economic growth drag effect under the constraints of resources and environment in the Yellow River Basin was carried out. Natural resources include three indicators: energy resources, water resources, and land resources. Environmental pollution indicators include industrial wastewater emissions, industrial SO_2_ emissions, and industrial smoke (powder) dust emissions, by drawing on Romer’s classic drag effect model and releasing the assumption of constant returns to scale, and expanding it, we can get:

(3) $$Y\left(t\right)=K{\left(t\right)}^{\alpha }E{\left(t\right)}^{\beta }W{\left(t\right)}^{\phi }R{\left(t\right)}^{\nu }B{\left(t\right)}^{\delta }S{\left(t\right)}^{\omega }D{\left(t\right)}^{\varphi }{\left[A\left(t\right)L\left(t\right)\right]}^{\theta }$$

Among them, $Y\left(t\right),\;K\left(t\right),\;E\left(t\right),\;W\left(t\right),\;R\left(t\right),\;B\left(t\right),\;S\left(t\right),\;D\left(t\right),\;A\left(t\right),\;L\left(t\right)$ respectively represent the GDP, capital stock, energy resources, water resources, land resources, and industrial wastewater discharge of each city in year t, industrial sulfur dioxide emissions, industrial smoke (dust) emissions, technological progress, and labor input. The product of A(t), L(t) represents the effective labor, α, β, ϕ, ν, δ, ω, φ, θ respectively represent the coefficient of elasticity of regional GDP, capital stock, energy resources, water resources, land resources, industrial wastewater emissions, industrial SO_2_ emissions, industrial smoke and dust emissions, and the effective labor.

Derived from the expansion of (3), taking the logarithm of both sides, we can get:

(4) $$\ln Y\left(t\right)=\alpha \ln K\left(t\right)+\beta \ln E\left(t\right)+\phi \ln W\left(t\right)+\nu \ln R\left(t\right)+\delta \ln B\left(t\right)+\omega \ln S\left(t\right)+\varphi \ln D\left(t\right)+\theta \left[\ln A\left(t\right)+\ln L\left(t\right)\right]$$

At this time, this article uses $g_{X}\left(t\right)$ to represent the growth rate of variable X, and the time derivative of the variables on both sides of Equation (4) is calculated, and the economic growth equation is:

(5) $$g_{Y}\left(t\right)=\alpha g_{K}\left(t\right)+\beta g_{E}\left(t\right)+\phi g_{W}\left(t\right)+\nu g_{R}\left(t\right)+\delta g_{B}\left(t\right)+\omega g_{S}\left(t\right)+\varphi g_{D}\left(t\right)+\theta \left[g_{A}\left(t\right)+g_{L}\left(t\right)\right]$$

Under the balanced growth path, this article assumes $g_{Y}\left(t\right)=g_{K}\left(t\right)$, $\dot{L}\left(t\right)=nL\left(t\right)$, $\dot{A}\left(t\right)=gA\left(t\right)$. That is, assuming that the growth rate of regional GDP is equal to the growth rate of capital stock, the labor growth rate is n, and the technological progress growth rate is g. This article believes that at a certain level of economic development, natural resources, and environmental pollution will restrict or drag economic growth to a certain extent. Due to the non-renewable nature of energy resources, energy consumption will rise, assuming that the short-term energy reserves will not. If changes occur, the rate of use will gradually decrease, which may also have a restraining effect on economic growth. Considering resource and environmental constraints, suppose that the rate of energy resource consumption is *e*, the rate of water consumption is *w*, the rate of land resource consumption is *r*, the growth rate of industrial wastewater discharge is *b*, the growth rate of industrial SO_2_ emissions is *s*, and the growth rate of industrial smoke and dust emissions is *d*.

Then, there is

(6) $$\begin{array}{l} \dot{E}\left(t\right)=-eE\left(t\right),\;e>0\\ \dot{W}\left(t\right)=-wW\left(t\right),\;w>0\\ \dot{R}\left(t\right)=-rR\left(t\right),\;r>0\\ \dot{B}\left(t\right)=-bB\left(t\right),\;b>0\\ \dot{S}\left(t\right)=-sS\left(t\right),\;s>0\\ \dot{D}\left(t\right)=-dD\left(t\right),\;d>0 \end{array}$$

Bringing Equation (6) into Equation (5), and arrange to get

(7) $$g_{Y}\left(t\right)=\frac{\beta e+\phi w+\nu r+\delta b+\omega s+\varphi d+\theta \left(g+n\right)}{1-\alpha }$$

In economic theory, the level of economic growth can be measured by the growth rate of output per capita. Based on Equation (7), the economic output of the constrained model can be derived as:

(8) $$\begin{array}{l} g_{Y/L}\left(t\right)=g_{Y}\left(t\right)-g_{K}\left(t\right)=\frac{\beta e+\phi w+\nu r+\delta b+\omega s+\varphi s+\theta \left(g+n\right)}{1-\alpha }-n\\ =\frac{\beta e+\phi w+\nu r+\delta b+\omega s+\varphi s+\theta \left(g+n\right)-n\left(1-\alpha \right)}{1-\alpha } \end{array}$$

According to the definition of economic growth drag effect, the drag effect of natural resources and environmental pollution on economic growth is the difference between the unconstrained and restricted economic growth. Therefore, in the absence of natural resources and environmental pollution constraints, assuming that $W\left(t\right),\;R\left(t\right),$ $R\left(t\right),\;B\left(t\right),\;S\left(t\right),\;D\left(t\right)$ continue to grow at the same speed as the labor input n, substitute them into Equation (7), the economic output of the unconstrained model can be obtained as follows:

(9) $${\hat{g}}_{Y/L}\left(t\right)={\hat{g}}_{Y}\left(t\right)-{\hat{g}}_{K}\left(t\right)=\frac{\beta n+\phi n+\nu n+\delta n+\omega n+\varphi n+\theta \left(g+n\right)-n\left(1-\alpha \right)}{1-\alpha }$$

The drag effect of economic growth under resource and environmental constraints can be obtained by subtracting Equation (9) from Equation (8):

(10) $$Drag={\hat{g}}_{Y/L}\left(t\right)-g_{Y/L}\left(t\right)=\frac{\left(n-e\right)\beta +\left(n-w\right)\phi +\left(n-r\right)\nu }{1-\alpha }+\frac{\left(n-b\right)\delta +\left(n-s\right)\omega +\left(n-d\right)\varphi }{1-\alpha }$$

It can be seen from Equation (10) that the drag effect of economic growth is composed of the drag effect of natural resources and the drag effect of environmental pollution. Among them, $\frac{\left(n-e\right)\beta +\left(n-w\right)\phi +\left(n-r\right)\nu }{1-\alpha }$ is the drag effect of natural resources, and $\frac{\left(n-b\right)\delta +\left(n-s\right)\omega +\left(n-d\right)\varphi }{1-\alpha }$ is the drag effect of environmental pollution.

#### 2.2.2. Construction of a Panel Model for the Economic Growth Drag Effect of the Yellow River Basin

(1) Construction of classic panel model
  (11) $$\ln Y_{it}=\alpha_{0}+\alpha \ln K_{it}+\beta \ln E_{it}+\phi \ln W_{it}+\nu \ln R_{it}+\delta \ln B_{it}+\omega \ln S_{it}+\varphi \ln D_{it}+\theta \left(\ln A_{it}+\ln L_{it}\right)+\varepsilon_{it}$$

Among them, $Y_{it}$ represents the gross regional product; $K_{it}$ represents the capital stock, $E_{it},\;W_{it},\;R_{it},\;B_{it},\;S_{it},\;D_{it},\;A_{it},\;L_{it}$, respectively, indicate the total gas supply, total water resources, land resources, industrial wastewater emissions, industrial SO_2_ emissions, industrial smoke (dust) emissions, technological progress, and urban unit employment in the number of people, $\varepsilon_{it}$ is the random error term, i represents the i-th prefecture-level city, and t represents the t-th year.

(2) Spatial Durbin model construction

In order to accurately estimate the impact of energy resources and environmental pollution on the economic growth of the Yellow River Basin, the prevalent spatial correlation should be considered. The economic growth of each city in the Yellow River Basin is not only affected by the region but also by the neighboring prefecture-level cities. The traditional ordinary panel model ignores the influence of variables in the surrounding area, leading to biases in the estimation [37,38]. Therefore, the spatial Durbin model for analysis was introduced. The spatial Durbin model believes that the explained variables are affected by the spatial lags of the explained variables in the local area and related explanatory variables, and also affected by the explanatory variables in adjacent areas.

Before performing spatial analysis, a spatial weight matrix needs to be constructed. This article uses a binary adjacency spatial weight matrix, which represents the relationship between spatial objects that are adjacent to each other. The general adjacency standard is:

(12) $$W_{ij}=\left\{{}_{0,\;i\;\mathrm{and}\;j\;\mathrm{are}\;\mathrm{not}\;\mathrm{adjacent}}^{1,\;i\;\mathrm{and}\;j\;\mathrm{are}\;\mathrm{adjacent}}\right.$$

In Equation (12), $W_{ij}$ is the adjacent space weight matrix, i and j are adjacent prefecture-level cities, i = 1,2,…, 80; j = 1,2,…, 80; i=j or i≠j, Usually all diagonal elements of command W are 0, which is $W_{ii}=W_{jj}=0$.

Based on this, the Spatial Durbin Model (SDM) is established as follows:

(13) $$\begin{array}{l} \ln Y_{it}=\alpha_{0}+\rho W\ln Y_{it}+\alpha \ln K_{it}+\beta \ln E_{it}+\phi \ln W_{it}+\nu \ln R_{it}+\delta \ln B_{it}+\omega \ln S_{it}+\varphi \ln D_{it}\\ +\theta \left(\ln A_{it}+\ln L_{it}\right)+\alpha^{\prime }W\ln K_{it}+\beta^{{}^{\prime }}W\ln E_{it}+\phi^{\prime }W\ln W_{it}+\nu^{\prime }W\ln R_{it}+\delta^{\prime }W\ln B_{it}+\omega^{\prime }W\ln S_{it}\\ +\varphi^{\prime }W\ln D_{it}+\theta^{\prime }W\left(\ln A_{it}+\ln L_{it}\right)+\varepsilon_{it} \end{array}$$

Among them, $Y_{it}$ represents the regional GDP, W is the spatial weight matrix, The spatial lag variable $W\ln Y_{it}$ refers to the weighted sum of the regional GDP of geographically adjacent prefecture-level cities, ρ is the spatial lag coefficient, which measures the spatial spillover effect of geographically-adjacent prefecture-level cities, $K_{it}$ represents the capital stock, $E_{it},\;W_{it},\;R_{it},\;B_{it},\;S_{it},\;D_{it},\;A_{it},\;L_{it}$, respectively, represent the total gas supply, total water resources, land resources, industrial waste water emissions, industrial SO_2_ emissions, industrial smoke (dust) emissions, technological progress, and employment in urban units. $\varepsilon_{it}$ is the random error term, i represents the i-th prefecture-level city, and t represents the t-th year. At the same time, the spatial lag term of the explanatory variable is introduced as $W\ln K_{it},$ $W\ln E_{it},$ $W\ln W_{it},$ $W\ln R_{it},$ $W\ln B_{it},$ $W\ln S_{it},$ $W\ln D_{it}$, and its coefficient is $\alpha^{\prime },\;\beta^{\prime },\;\phi^{\prime },\;\nu^{\prime },\;\delta^{\prime },\;\omega^{\prime },\;\varphi^{\prime },\;\theta^{\prime }$.

## 3. Drag Effects Analysis and Discussion 

### 3.1. Three Types of Drag Effects in a Single Prefecture-Level City

**Figure 2:**
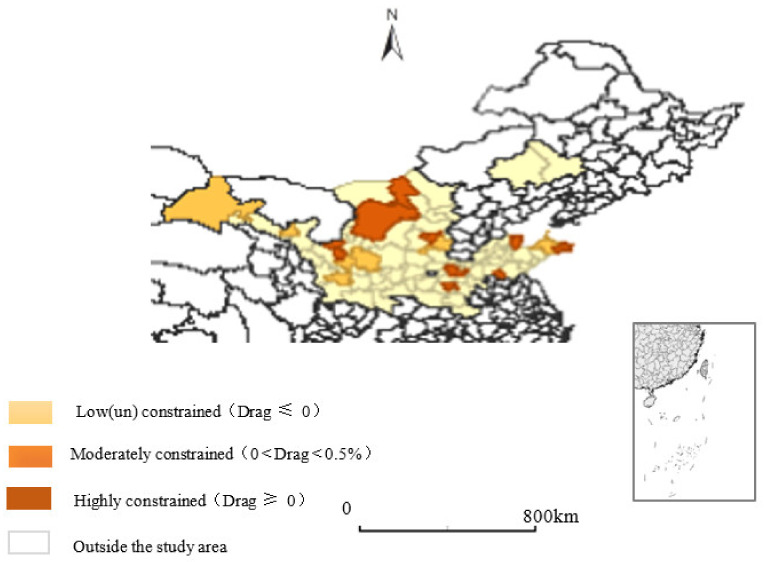
The distribution of economic growth drag effects in the Yellow River Basin.

In order to measure the growth drag effect that is caused by resource and environmental constraints, this paper selects the balanced short panel data of 80 prefecture-level cities in the Yellow River Basin from 2003 to 2018, and uses Romer’s classic drag effect model to expand and test regression. The city conducts cross-sectional regression to obtain the capital stock elasticity coefficient (α), total gas supply elasticity coefficient (β), water resource coefficient (φ), land resource elasticity coefficient (ν), and industrial wastewater discharge that are required in the drag efficiency calculation process elastic coefficient (δ), industrial SO_2_ emission elastic coefficient (ω), and the industrial smoke and dust elastic coefficient (ψ). In addition, the average annual growth rate of the labor force (n), the average annual growth rate of total gas supply (e), the average annual growth rate of water resources (w), the average annual growth rate of land resources (r), the average annual growth rate of industrial wastewater discharge (b), the average annual growth rate of industrial SO_2_ emissions (s), and the average annual growth rate of industrial smoke and dust (d) are substituted into Equation (9), and it can be obtained that the 80 prefecture-level cities in the Yellow River Basin from 2003 to 2018 are subject to resource and environmental constraints. The economic growth natural resource drag effect, environmental drag effect, and the total drag effect are shown in Table 2. In view of the fact that the Yellow River Basin spans the east, central, and western regions, and the regional economic development of the upper, middle, and lower reaches is significantly different, the following section will compare the effects of natural resources, environmental pollution, and economic growth in the Yellow River Basin for regional comparison and characteristic impact analysis(see Figure 2 for details).

On the basis of related research by Qin; et al. [39], the natural resource drag efficiency, environmental drag efficiency, and economic growth drag efficiency of each city in the Yellow River Basin are divided into low (un)constrained type (Drag ≤ 0), medium Constraint type (0 < Drag < 0.5%), and high constraint type (Drag ≥ 0.5%). It can be seen from Table 3 that in the drag efficiency model of prefecture-level cities in the Yellow River Basin.

### 3.2. Regional Differences in Natural Resource Drag Effects

The prefecture-level cities with low (un)constrained natural resource drag-efficiency accounted for 58.75% of the total sample size, medium-constrained type accounted for 22.5%, and high-constrained type accounted for 10%. Among them, 52 prefecture-level cities such as Lanzhou, Baiyin, and Wuwei are of low (un)constraint type, indicating that these areas are not constrained by natural resource tail effects. A total of 18 prefecture-level cities such as Jiayuguan, Jinchang, and Tianshui are medium-constrained; 10 prefecture-level cities such as Zhongwei, Baotou, and Wuhai are high-constrained. It can be seen that the low (un)constrained type of natural resources in the lower reaches of the Yellow River is the most significant, and the medium-constraint and high-constraint types are the most significant in the middle reaches of the Yellow River

### 3.3. Regional Differences in Environmental Pollution Drag Effects

The prefecture-level cities with low (un)constrained environmental pollution drag effects accounted for 75% of the total sample size, medium-constrained types accounted for 17.5%, and high-constrained types accounted for 7.5%. Specifically, 60 cities such as Lanzhou, Jiayuguan, and Wuwei are of the low (un)constrained type, 14 prefecture-level cities such as Jinchang, Baiyin, and Tianshui are of the medium-constrained type, and 6 prefecture-level cities such as Qingyang, Wuzhong, and Baotou are high-constrained type. It can be seen that the low (un)constrained type of environmental pollution in the middle reaches of the Yellow River is the most significant, and the medium-constraint and high-constraint types of the upper reaches of the Yellow River are the most significant.

### 3.4. Regional Differences in Total Drag Effects

The total drag effects were low (un)constrained prefecture-level cities accounted for 78.75% of the total sample size, medium-constrained cities accounted for 8.75%, and highly-constrained cities accounted for 12.5%. A total of 63 prefecture-level cities such as Lanzhou, Jiayuguan, and Baiyin are of low (un)constrained type, 7 prefecture-level cities such as Jinchang, Tianshui, Jiuquan are of the medium-constrained type, and 10 prefecture-level cities such as Zhongwei, Taiyuan and Baotou are of high-constrained type. It can be seen that the low (un)constrained type of environmental pollution in the middle reaches of the Yellow River is the most significant, and the medium-constrained and high-constrained types of the upper reaches of the Yellow River are the most significant. 

## 4. Spatial Effect Results and Discussion

### 4.1. Analysis of the Results of Classic Panel Regression and Spatial Regression of Drag Effects

First, the classical panel model is used to estimate the parameters, and the first step is to perform the panel unit root test. The LLC (Levin, Lin, and Chu) test shows that the adjusted $t_{\delta }^{*}$ statistic (Adjusted t*) is −4.8568, which is significantly negative. The IPS (Im, Pesaran, and Shin) test shows that the $\bar{t}$ statistic is −7.4178, which is less than the critical value of −1.730 at the 1% significance level. Therefore, the null hypothesis of the panel unit root is rejected. In the second step, the Hossman test is performed. The Hausman effect shows that the Hausman test value is 56.60 and the *p*-value is 0.0000. Therefore, the null hypothesis of random effects is strongly rejected, and the fixed effects model should be used for this model. Secondly, the model with spatial effects is tested. According to the LR (likelihood ratio) test, the statistics are 897.1156 and 649.6271, respectively, under the fixed effects in time and space, and they are significant at the 1% level. The results of Hausman test show the *p*-value is 0.0000, so the null hypothesis of random effects is strongly rejected. As such, this article chooses the dual fixed effects model. Finally, in order to ensure the robustness of model estimation, this paper conducted Wald test and LR test on SAR (spatial autoregressive model), SEM (spatial error model), and SDM (spatial Durbin model) to judge the rationality of SDM. The results show that the LR statistics and Wald statistics of SAR and SEM are significantly greater than 0, and the *p*-value is 0.0000. The null hypothesis is rejected, indicating that the SDM cannot be degenerated into SAR or SEM, and the R-square and log-likelihood values of SDM are both better than SAR and SEM, to a certain extent, indicate that the SDM model has the best fitting effect. Therefore, this paper intends to select the SDM with dual fixed effects to estimate the regression parameters and to decompose its direct and indirect effects to clarify the direct and indirect effects of each element on the regional economic growth. The regression results are shown in Table 4.

From the regression results of the classic panel model in Table 5, it can be seen that the elasticity coefficients of capital stock, energy resources, water resources, land resources, industrial wastewater emissions, industrial SO_2_ emissions, and industrial smoke (dust) emissions are below the 1% level. Obviously, the elasticity of capital stock, energy resources, water resources, and land resources is significantly positive, indicating that these factors have a significant boost to economic growth. In addition, the elasticity coefficients of industrial wastewater emissions and industrial waste gas emissions are significantly negative, indicating that the Yellow River Basin Economic growth is obviously restricted and inhibited by wastewater and waste gas pollution. Among them, the effect of effective labor on the economic growth of the Yellow River is not significant, and the effect of industrial soot emission on the economic growth is significantly positive. Since the classical panel model does not consider the impact of spatial effects, we will see whether the answer can be found from the SDM regression estimation results.

Table 4 shows the direct effect, indirect effect, and total effect results of the capital stock, effective labor, energy resources, water resources, land resources, industrial wastewater discharge, industrial SO_2_ discharge, and industrial smoke (powder) dust discharge on the economic growth of the Yellow River Basin.

With respect to direct effect, the capital stock, water resources, land resources, and the industrial SO_2_ emissions in this region all have a significant positive effect on the regional economic development, The Yellow River Basin is relatively short of water resources and suffers from serious soil erosion. However, the basic demand for water resources and land resources in the current stage of economic development of the Yellow River Basin is large. Therefore, the vigorous investment of the two will certainly promote economic growth to a certain extent. The large amount of industrial SO_2_ emissions is related to the vigorous development of heavy chemical industries such as chemical raw materials and chemical fiber manufacturing in the Yellow River Basin. Energy resources and industrial wastewater discharge have a significant negative effect on the economic development of the region. The characteristics of energy resources are non-renewable. As the utilization of energy resources intensifies, the available energy resources drop sharply. At this time, the extraction and processing of energy resources largely determines the level of economic development in the region 

With respect to the indirect effects, water resources and industrial SO_2_ emissions in adjacent areas have obvious positive effects on the economic growth of the region The richer the water resources in the adjacent areas, the more significant the economic growth in the region This shows that the Yellow River Basin is short of water resources, and economic development is limited to a certain extent by water resources, In addition, the water resource has fluidity, and the region is vulnerable to the influence of neighboring areas, especially the adjacent areas in the upper and lower reaches of the river. The upstream water resource has a great influence on the prefecture-level cities in the lower reaches. The larger the industrial SO_2_ emissions, the more obvious the environmental pollution that is caused to the region. Relevant industrial enterprises may go to neighboring prefecture-level cities to develop under the influence of relevant governance policies in the region, thereby promoting the economic development of neighboring prefecture-level cities. The industrial wastewater discharge in adjacent regions have obvious negative effects on the economic growth of the region. Industrial wastewater is an important cause of water pollution. Naturally, the decline of river water quality and total water resources in adjacent prefecture-level cities will affect the domestic and industrial water use in the region. 

With respect to the total effect, effective labor, water resources, land resources, industrial wastewater discharge, and industrial SO_2_ discharge have significant effects on the economic growth of the Yellow River Basin at the level of 10%. The elastic coefficients of effective labor and industrial wastewater discharge are significantly negative, and the elastic coefficients of water resources, land resources, and industrial SO_2_ emissions are significantly positive. In addition, capital stock, energy resources, and industrial smoke (powder) dust emissions have no significant effect on economic growth. Obviously, whether the elasticity of each element in the total effect is significant depends on the size and significance of the elasticity of each element in the direct effect and indirect effect, so it is not repeated here.

### 4.2. Analysis of the Drag Effect Results under the Classic Panel Model and SDM

As shown in Table 5, the total drag effect of economic growth in the Yellow River Basin is −0.8347 through the classic panel model estimation. The average growth rate increased by 0.8347%. It is estimated that the drag effect of natural resources is −0.7204, and the drag effect of environmental pollution is −0.1143. It can be seen that from 2003 to 2018, the natural resources of the Yellow River Basin are still a positive incentive for the economy of the Yellow River Basin, and the constraints of environmental pollution on economic growth are gradually reduced. Thanks to the advancement of measures such as resource conservation, environmental friendliness, energy conservation, and emission reduction in recent years, and the practice of the concept of green development. From the perspective of the coefficient of economic factors, the capital stock increased by 1%, and the economy of the Yellow River Basin increased by 0.7670%. Energy resources, water resources, land resources, and industrial smoke (powder) dust emissions increased by 1%, and the economy increased by 0.0168%, 0.1480%, 0.1700%, and 0.0712%, respectively. The industrial wastewater discharge and industrial SO_2_ discharge increased by 1%, and the economy decreased by 0.1100% and 0.0542%, respectively. This shows that the Yellow River Basin is still an extensive economic growth model that is dominated by capital investment. Consumption has an increasingly significant impact on the environment, and the arrival of resource and environmental carrying capacity thresholds is likely to have an inhibitory effect on economic growth.

An observation from Table 5 finds that the conclusions that are drawn through the SDM calculations are completely opposite to those that are drawn by the classic panel model. After incorporating the spatial effects, we find that the total drag effect of economic growth in the Yellow River Basin is 0.0137, which shows that the average annual economic growth rate of the Yellow River Basin has dropped by 0.0137% under the constraints of natural resources and environmental pollution. The drag effect of natural resources is 0.0095, and the drag effect of environmental pollution is 0.0042, indicating that both natural resources and environmental pollution have restrained the economic growth of the Yellow River Basin. The reason for the difference between the results from the classic panel estimation is that the economic factors of prefecture-level cities in the Yellow River Basin have a certain spatial-dependence effect and spatial spillover effect, that is, the economic factors of neighboring prefecture-level cities will have an impact on the economic growth of the prefecture-level city. This leads to discrepancies in the estimated results. From the perspective of the economic factor coefficient, the capital stock increased by 1%, and the economy of the Yellow River Basin increased by 0.6783%. Energy resources and industrial wastewater discharge increased by 1%, and the economy decreased by 0.0041% and 0.0412%, respectively. Water resources, land resources, industrial SO_2_ emissions, and industrial smoke (powder) dust emissions increased by 1%, and the economy increased by 0.0240%, 0.0752%, 0.0443%, and 0.0057%, respectively.

Decomposing the spatial effect of the SDM, the total drag effect of the direct effect is 0.0248, the total drag effect of the indirect effect is −0.0387. The total drag effect of the direct effect is much larger than the total drag effect of the indirect effect, and the total drag effect of the direct effect is significantly positive. The direct effects of natural resource drag effects and environmental pollution drag effects are also significantly positive. It indicates that the constraints of resources and environmental pollution in the region strongly restrict economic growth in the region, while the total drag effects of indirect effects are significantly negative, and the indirect effects of natural resource drag effects and environmental pollution drag effects is also significantly negative, indicating that the resources and environmental pollution of neighboring prefecture-level cities have a promoting effect on the economic development of the region. Observing the direct and indirect effects of economic factor coefficients, it is found that the direct effect coefficient of capital stock is 0.6480, indicating that the capital stock increased by 1%, the economy of this prefecture-level city will grow by 0.6480%, and the indirect effect coefficient of capital stock is −0.4456, indicating that the neighboring regions the capital stock has a restraining effect on the local economy. The denser the capital in neighboring areas, the less capital flows to the prefecture-level city. The direct and indirect effect coefficients of energy resources and industrial wastewater discharge are significantly negative, indicating that regardless of the factors in the region or neighboring regions, these two factors have a negative impact on the economic growth of the region. The direct and indirect effect coefficients of water resources, land resources, industrial SO_2_ emissions, and industrial smoke and dust emissions are significantly positive, that is, it has a positive impact on the economic growth of the region.

### 4.3. The Economic Growth Drag Effect Results of the Upper, Middle, and Lower Reaches of the Yellow River Basin

First, the models with spatial effects in the upper, middle, and lower reaches of the Yellow River are tested. Through the Hausman test, the fixed-effect model is selected for the upper and lower reaches of the Yellow River, the random-effects model is selected for the middle reaches of the Yellow River, and the SDM is selected through the robust test. Table 6 shows the SDM estimation results and the effect of decomposition of the upper, middle, and lower reaches of the Yellow River Basin. For the upper reaches of the Yellow River, the direct effects of capital stock and land resources on the economic growth of the upper reaches of the Yellow River are significantly positive. The direct effect of effective labor, energy resources, and industrial wastewater discharge on the economic growth of the upper reaches of the Yellow River is significantly negative. The indirect effect of land resources in adjacent areas on the economic growth of the upper reaches of the Yellow River is significantly positive. The indirect effects of capital stock, effective labor, and industrial wastewater discharge in adjacent areas on economic growth of the upper reaches of the Yellow River are significant. For the middle reaches of the Yellow River, the direct effects of capital stock, energy resources, water resources, land resources, and industrial smoke and dust emissions on the economic growth are significantly positive, and the direct effects of industrial SO_2_ emissions on the economic growth of the middle reaches of the Yellow River are significantly negative. The indirect effects of energy resources and industrial smoke and dust emissions in neighboring regions on the economic growth of the middle reaches of the Yellow River are significantly positive, and the indirect effects of capital stock and industrial SO_2_ emissions in neighboring regions on the economic growth of the middle reaches of the Yellow River are significantly negative. For the lower reaches of the Yellow River, the direct effects of water resources and industrial wastewater discharge on the economic growth are significantly positive, and the direct effects of capital stock, effective labor, and industrial smoke and dust emissions on the economic growth are significantly negative. The indirect effect of growth is significantly positive and the indirect effects of energy resources and industrial wastewater discharge on the economic growth of the lower Yellow River are significantly negative.

According to Table 7, the economic growth drag effect results of the upper, middle, and lower reaches of the Yellow River Basin. From the total effect of the SDM, the total drag effect of economic growth in the upper reaches of the Yellow River is 0.0156, indicating that natural resources and environmental pollution have an inhibitory effect on economic growth in the upper reaches of the Yellow River. The total drag effect of economic growth in the middle reaches of the Yellow River is −0.1786, and the downstream is −0.0111, indicating that for the middle and lower reaches of the Yellow River, natural resources and environmental pollution do not restrict its economic development, but promote it. In terms of the direct effects, the total drag effect of economic growth in the upper reaches of the Yellow River is 0.0021, indicating that natural resources and environmental pollution have a restraining effect on economic growth. The total drag effect of economic growth in the middle reaches of the Yellow River is −0.2111, and the downstream is −0.0035, which is also a promotion. From the perspective of indirect effects, the total drag efficiency constraint of the upper and middle reaches of the Yellow River is negative, that is, the economic factors of the neighboring regions have a positive effect on the economic development of the region, and the total drag efficiency constraint of the lower Yellow River is positive, indicating that the economic factors of the neighboring regions have a positive effect on the region at this stage. Economic development has a certain restraining effect.

### 4.4. Further Discussion

By summarizing further findings, it is found that one of the reasons why the Yellow River Basin is restricted by resources and environmental pollution is the conflict between the resource development and utilization and ecological environmental protection. The river basin is rich in energy resources, but the uniqueness and fragility of the ecological environment determines that the development and utilization of resources are restricted. In addition, the non-renewable characteristics of energy resources will not be discovered in the short term. Energy resources mean that the rate of use of energy resources will decline, and the energy industry in the Yellow River Basin is relatively developed, and industrial development is obviously restricted by resource reserves, thereby inhibiting economic growth. Second, the shortage of water resources and the contradiction between supply and demand are more prominent. There is a shortage of water resources in the Yellow River Basin. Per capita water resources are less than 30% of the national average, but the efficiency of water resource development and use is as high as 80%, and heavy chemical industry pollution is serious. Water quality is also affected and there are structural contradictions in water use and extensive water use methods; it has not been fundamentally reversed and soil erosion is serious. The long-term natural and man-made factors have not only reduced the level of water resource utilization, but also reduced the effective land use area. As the most rigid constraint for the development of the Yellow River Basin, water resources must be optimized for water resources allocation, and the source governance can break through this constraint. Third, there is a contradiction between the environmental carrying capacity and economic development needs. The Yellow River Basin is mainly based on energy and heavy chemical industries, these industries have great environmental pollution and damage, and the problems of the three industrial wastes are prominent. The Yellow River Basin is also known as the energy basin. Various heavy and chemical industries are concentrated in the basin along the Yellow River. In addition, the Yellow River Basin itself has a fragile ecological environment and a limited carrying capacity. Industrial wastewater, industrial waste gas, and industrial smoke and dust cannot be effectively absorbed and converted. Therefore, the environmental capacity also restricts the economic development of the Yellow River Basin.

## 5. Conclusions and Policy Recommendations

### 5.1. Main Conclusions

This paper uses the panel data of 80 prefecture-level cities in the Yellow River Basin from 2003 to 2018 and extends Romer’s classic drag effect model to construct an economic growth model under the constraints of resources and environment in the Yellow River Basin. It measured the drag effect of economic growth in the Yellow River Basin under constraints and unconstrained conditions and carried out regional spatial difference analysis. The main conclusions are as follows:

(1) The total drag effect of economic growth of a single prefecture-level city is divided into three types: low (un)constrained (Drag ≤ 0), medium-constrained (0 < Drag < 0.5%), and high-constrained (Drag ≥ 0.5%). The low (un)constrained type includes 63 prefecture-level cities such as Lanzhou, Jiayuguan, and Baiyin; the medium-constrained type includes 7 prefecture-level cities such as Jinchang, Tianshui, and Jiuquan; and the high-constrained type includes 10 prefecture-level cities such as Zhongwei, Taiyuan, and Baotou. It is found that the medium-constrained type is the most significant in the total drag effects of the prefecture-level cities in the upper reaches of the Yellow River, and the high-constrained type is the most significant in the lower reaches of the Yellow River. 

(2) Through the classical panel model and the SDM model, the overall economic growth drag effect of the Yellow River Basin is calculated, and it is found that the overall economic growth drag effect of the Yellow River Basin that was obtained by the classic panel model without adding the spatial effect is significantly negative. It indicates that the resources and the environment have a significant impact on the Yellow River Basin. This is consistent with the conclusion that the average total drag effect of the 80 prefecture-level cities above is negative, while the total drag effect that was obtained by the SDM model with spatial effect is significantly positive, and the total drag effect of the overall economic growth in the Yellow River Basin has changed from unconstrained to moderately constrained.

(3) By analyzing the total drag effect of economic growth in the upper, middle, and lower reaches of the Yellow River, it is found that the resources and the environment have a certain drag effect on the economic growth of the upper reaches of the Yellow River, while promoting the middle and lower reaches of the Yellow River. The total drag effect of the direct effect and the total drag effect of the total effect are the same. The total drag effect of indirect effects is expressed as positive constraints on economic growth in the lower reaches of the Yellow River, and positive incentives in the upper and middle reaches of the Yellow River.

### 5.2. Policy Suggestion

The above conclusions have important policy implications for the Yellow River Basin to surmount the constraints of natural resources and environmental pollution to achieve coordinated economic development in the basin:

(1) The government should ensure the sustainable and efficient use of resources and cultivate new drivers of green development. Relevant apartments should choose suitable green technologies for ecological transformation and promote the soft connection of resources and industries in various regions of the Yellow River Basin. It helps to break the administrative protection boundaries and trade barriers of various regions.

(2) All regions should undertake joint prevention and control of environmental pollution to achieve greater protection of the ecological environment of the river basin, establish a government-led and enterprise-oriented horizontal ecological protection, and compensation mechanism along the Yellow River basin [40].

(3) Provinces should pay attention to the spatial difference between resource planning and environmental regulation policies. All provinces should comprehensively promote the cross-regional resource allocation and cross-regional ecological environment protection mechanism in the Yellow River Basin.

## Figures and Tables

**Table 1:** Descriptive statistical results of main variables.

Main Variable	Unit	Sample Size	Mean	Standard Deviation	Minimum	Maximum
GDP (*Y*)	Ten thousand yuan	1280	10,996,580	12,720,747	210,687	92,433,263
Capital stock (*K*)	Ten thousand yuan	1280	10,840,525	12,443,174	207,025	82,804,866
Effective labor (*AL*)	Hour	1280	3,501,236	3,405,758	323,281	23,001,356
Energy resources (*E*)	Ten thousand cubic meters	1280	17,076	35,771	1	745,182
Water resources (*W*)	Ten thousand cubic meters	1280	184,266	245,450	205	2,279,600
Land Resources (*R*)	Square kilometers	1280	90	85	3	658
Industrial wastewater discharge (*B*)	Ten thousand tons	1280	5110	4799	99	28,191
Industrial SO_2_ emissions (*S*)	Ton	1280	65,786	55,031	633	337,164
Industrial smoke (dust) emissions (*D*)	Ton	1280	35,792	96,929	56	3,153,822

**Table 2:** The economic growth effect of 80 prefecture-level cities in the Yellow River Basin from 2003 to 2018.

Prefecture-Level City	Taiyuan	Datong	Yangquan	Changzhi	Jincheng	Shuozhou	Jinzhong	Yuncheng	Xinzhou	Linfen
Natural resource drag effect (%)	0.4927	0.0716	0.0060	−0.7223	−0.4131	−0.0095	0.2236	0.0931	−0.6147	−0.3503
Environmental pollution drag effect (%)	0.0252	−0.7414	−0.0088	−0.3926	−0.3090	−0.5690	0.2258	−0.0976	0.0731	−0.0153
Total drag effect (%)	0.5179	−0.6698	−0.0029	−1.1149	−0.7221	−0.5785	0.4495	−0.0045	−0.5417	−0.3655
Prefecture-level city	Luliang	Hohhot	Baotou	Wuhai	Chifeng	Tongliao	Ordos	Bayannaoer	Wulanchabu	Jinan
Natural resource drag effect (%)	−1.0134	0.1691	6.1147	2.2561	−0.0813	−0.0775	0.9787	0.6402	0.0553	−0.7025
Environmental pollution drag effect (%)	−0.0380	−0.2319	1.7951	−0.4369	−0.4860	−0.2778	−0.0444	−3.4039	−0.4091	0.2631
Total drag effect (%)	−1.0514	−0.0628	7.9098	1.8192	−0.5673	−0.3553	0.9343	−2.7637	−0.3538	−0.4394
Prefecture-level city	Qingdao	Zibo	Zaozhuang	Dongying	Yantai	Weifang	Jining	Tai’an	Weihai	Rizhao
Natural resource drag effect (%)	−1.8576	−0.0581	1.2073	−0.8763	0.4128	−0.3334	−0.8016	−0.6489	0.9906	−1.4586
Environmental pollution drag effect (%)	−1.8972	−0.5157	−0.2396	4.4830	−0.1619	−0.2317	−0.5716	−0.8008	−0.3424	−0.1671
Total drag effect (%)	−3.7548	−0.5738	0.9678	3.6067	0.2510	−0.5651	−1.3733	−1.4497	0.6482	−1.6257
Prefecture-level city	Laiwu	Linyi	Dezhou	Liaocheng	Binzhou	Heze	Zhengzhou	Kaifeng	Luoyang	Pingdingshan
Natural resource end effect (%)	−0.2070	−1.3444	−0.8130	−1.8578	−0.5795	−0.7496	−0.2396	−0.6379	0.8049	−0.1847
Environmental pollution drag effect (%)	−0.4381	0.1050	−0.3704	−0.0429	−0.3331	−0.1279	−0.0860	−0.0940	−1.2916	−0.3614
Total drag effect (%)	−0.6451	−1.2395	−1.1833	−1.9008	−0.9126	−0.8775	−0.3256	−0.7318	−0.4867	−0.5461
Prefecture-level city	Anyang	Hebi	Xinxiang	Jiaozuo	Puyang	Xuchang	Luohe	Sanmenxia	Nanyang	Shangqiu
Natural resource drag effect (%)	−4.3472	−1.1519	−6.6325	−0.0195	−0.8639	3.9397	−0.7620	−1.1178	−0.8147	0.1011
Environmental pollution drag effect (%)	1.0026	0.1850	10.1779	−0.6514	−0.5565	−1.4055	−0.5307	−0.3594	−0.4254	−0.5957
Total drag effect (%)	−3.3446	−0.9669	3.5454	−0.6708	−1.4204	2.5342	−1.2927	−1.4771	−1.2400	−0.4946
Prefecture-level city	Xinyang	Zhoukou	Zhumadian	Xi’an	Tongchuan	Baoji	Xianyang	Weinan	Yan’an	Hanzhong
Natural resource drag effect (%)	−1.0078	−0.4242	0.1748	0.0980	−0.9570	13.6347	0.3095	−3.9266	−0.8350	0.0514
Environmental pollution drag effect (%)	0.0345	−0.1908	−0.2540	−0.2063	−0.2357	−61.5083	−0.6221	0.0346	−0.0935	−0.1571
Total drag effect (%)	−0.9733	−0.6150	−0.0791	−0.1082	−1.1927	−47.8737	−0.3126	−3.8921	−0.9285	−0.1057
Prefecture-level city	Yulin	Ankang	Shangluo	Lanzhou	Jiayuguan	Jinchang	Silver	Tianshui	Wuwei	Zhangye
Natural resource drag effect (%)	−1.6976	−0.1051	−1.3425	−0.1793	0.0190	0.2086	−0.7379	0.0748	−0.4381	0.0154
Environmental pollution drag effect (%)	−0.5067	−0.6294	−0.1335	−0.4824	−0.4174	0.1237	0.2605	0.1535	−0.0879	−0.1803
Total drag effect (%)	−2.2043	−0.7344	−1.4761	−0.6617	−0.3985	0.3323	−0.4775	0.2283	−0.5260	−0.1649
Prefecture-level city	Pingliang	Jiuquan	Qingyang	Dingxi	Longnan	Yinchuan	Shizuishan	Wu Zhong	Guyuan	Zhongwei
Natural resource drag effect (%)	−1.4086	0.1662	−0.1365	−0.3527	−0.3279	−0.5504	−0.1209	−8.2142	−0.2728	1.9752
Environmental pollution drag effect (%)	−0.3701	0.1197	0.5258	−0.8327	−0.1287	0.0366	−0.0427	0.6612	0.4016	−0.8247
Total drag effect (%)	−1.7786	0.2859	0.3893	−1.1855	−0.4566	−0.5137	−0.1635	−7.5530	0.1288	1.1505

**Table 3:** Drag effect model of the segmented basins of the Yellow River Basin.

Drag Effect Mode	Segmented Basins	Natural Resource Drag Effect	Environmental Pollution Drag Effect	Total Drag Effect
Low (un) constrained (Drag ≤ 0)	Upper Yellow River	Lanzhou, Baiyin, Wuwei, Pingliang, Qingyang, Dingxi, Longnan, Yinchuan, Shizuishan, Wuzhong, Guyuan (11)	Lanzhou, Jiayuguan, Wuwei, Zhangye, Pingliang, Dingxi, Longnan, Shizuishan, Zhongwei (9)	Lanzhou, Jiayuguan, Baiyin, Wuwei, Zhangye, Pingliang, Dingxi, Longnan, Yinchuan, Shizuishan, Wuzhong City (11)
	Middle Yellow River	Changzhi, Jincheng, Shuozhou, Xinzhou, Linfen, Luliang, Tongchuan, Weinan, Yan’an, Yulin, Ankang, Shangluo, Chifeng, Tongliao (14)	Datong, Yangquan, Changzhi, Jincheng, Shuozhou, Yuncheng, Linfen, Luliang, Hohhot, Wuhai, Chifeng, Tongliao, Ordos, Bayanzhuoer, Ulanchabu, Xi’an, Tongchuan, Baoji, Xianyang, Yan’an, Hanzhong, Yulin, Ankang, Shangluo (24)	Datong, Yangquan, Changzhi, Jincheng, Shuozhou, Yuncheng, Xinzhou, Linfen, Luliang, Hohhot, Chifeng, Tongliao, Bayanzhuoer, Ulanchabu, Xi’an, Tongchuan, Baoji, Xianyang, Weinan, Yan’an, Hanzhong, Yulin, Ankang, Shangluo (24)
	Lower Yellow River	Jinan, Qingdao, Zibo, Dongying, Weifang, Jining, Taian, Rizhao, Laiwu, Linyi, Dezhou, Liaocheng, Binzhou, Heze, Zhengzhou, Kaifeng, Pingdingshan, Anyang, Hebi, Xinxiang, Jiaozuo, Puyang, Luohe, Sanmenxia, Nanyang, Xinyang, Zhoukou (27)	Qingdao, Zibo, Zaozhuang, Yantai, Weifang, Jining, Taian, Weihai, Rizhao, Laiwu, Dezhou, Liaocheng, Binzhou, Heze, Zhengzhou, Kaifeng, Luoyang, Pingdingshan, Jiaozuo, Puyang, Xuchang, Luohe, Sanmenxia, Nanyang, Shangqiu, Zhoukou, Zhumadian (27)	Jinan, Qingdao, Zibo, Weifang, Jining, Taian, Rizhao, Laiwu, Linyi, Dezhou, Liaocheng, Binzhou, Heze, Zhengzhou, Kaifeng, Luoyang, Pingdingshan, Anyang, Hebi, Jiaozuo, Puyang, Luohe, Sanmenxia, Nanyang, Xinyang, Shangqiu, Zhoukou, Zhumadian (28)
Moderately constrained (0 < Drag < 0.5%)	Upper Yellow River	Jiayuguan, Jinchang, Tianshui, Zhangye, Jiuquan (5)	Jinchang, Baiyin, Tianshui, Jiuquan, Yinchuan, Guyuan (6)	Jinchang, Tianshui, Jiuquan, Qingyang, Guyuan (5)
	Middle Yellow River	Hohhot, Ulan Chabu, Xi’an, Xianyang, Hanzhong, Taiyuan, Datong, Yangquan, Jinzhong, Yuncheng (10)	Taiyuan, Jinzhong, Xinzhou, Weinan (4)	Jinzhong (1)
	Lower Yellow River	Yantai, Shangqiu, Zhuma (3)	Jinan, Linyi, Hebi, Xinyang (4)	Yantai (1)
Highly constrained (Drag ≥ 0.5%)	Upper Yellow River	Zhongwei (1)	Qingyang, Wu Zhong (2)	Zhongwei (1)
	Middle Yellow River	Baotou, Wuhai, Ordos, Bayannaoer, Baoji (5)	Baotou (1)	Taiyuan, Baotou, Wuhai, Ordos (4)
	Lower Yellow River	Zaozhuang, Weihai, Luoyang, Xuchang (4)	Dongying, Anyang, Xinxiang (3)	Zaozhuang, Dongying, Weihai, Xinxiang, Xuchang (5)

**Table 4:** Classic panel model and SDM estimation results and their effect decomposition.

Variable	Classic Panel Model	SDM	Direct Effect	Indirect Effect	Total Effect	Weight Variable	SDM
lnK	0.767 ***	0.6873 ***	0.6480 ***	−0.4456 *	0.2024	WlnK	−6.4610 ***
	(29.25)	(6.6800)	(6.6400)	(−1.8500)	(0.8500)		(−5.3800)
lnAL	0.0415	0.0316 **	0.0016	−0.3040 ***	−0.3030 **	WlnAL	−0.0992 ***
	(1.59)	(2.0450)	(0.0780)	(−2.9890)	(−2.6560)		(−3.7670)
lnE	0.0168 ***	−0.0041 *	−0.0060 *	−0.0224	−0.0284	WlnE	−0.0022
	(4.11)	(−1.75)	(−1.92)	(−1.32)	(−1.47)		(−0.5160)
lnW	0.148 ***	0.0240 **	0.0471 ***	0.2410 ***	0.2880 **	WlnW	0.0395 **
	(7.62)	(2.2450)	(3.1180)	(2.7370)	(2.8780)		(2.0250)
lnR	0.170 ***	0.0752 ***	0.1200 ***	0.4580 ***	0.5770 ***	WlnR	0.0543 *
	(5.82)	(4.7780)	(5.0270)	(3.1780)	(3.5350)		(1.7450)
lnB	−0.110 ***	−0.0412 ***	−0.0860 ***	−0.4670 ***	−0.5530 ***	WlnB	−0.0833 ***
	(−7.05)	(−4.7450)	(−7.1000)	(−6.3189)	(−6.6670)		(−5.1170)
lnS	−0.0542 ***	0.0443 ***	0.0667 ***	0.2330 ***	0.3000 ***	WlnS	0.0235 *
	(−4.80)	(5.9260)	(5.7270)	(3.7190)	(4.1450)		(1.7560)
lnD	0.0712 ***	0.0057	0.0102	0.0487	0.0589	WlnD	0.0070
	(6.79)	(0.9290)	(1.2560)	(0.9670)	(1.0350)		(0.6001)

Note: ***, **, * means passing the significance test at the level of 1%, 5%, and 10%, respectively. The values in () are statistical values, the same below.

**Table 5:** The drag effect results under the classic panel model and SDM.

Variable	Classic Panel Model	SDM	Direct Effect	Indirect Effect	Total Effect
Capital stock elasticity coefficient (α)	0.767	0.6873	0.648	−0.4456	0.2024
Energy resource elasticity coefficient (β)	0.0168	−0.0041	−0.006	−0.0224	−0.0284
Water resource coefficient (φ)	0.148	0.024	0.0471	0.241	0.288
Elasticity coefficient of land resources (ν)	0.17	0.0752	0.12	0.458	0.577
Industrial wastewater discharge elasticity coefficient (δ)	−0.11	−0.0412	−0.086	−0.467	−0.553
Industrial SO_2_ emission elasticity coefficient (ω)	−0.0542	0.0443	0.0667	0.233	0.3
Industrial smoke emission elasticity coefficient (ψ)	0.0712	0.0057	0.0102	0.0487	0.0589
Natural resource end effect (%)	−0.7204	0.0095	0.0166	−0.0659	0.439
Environmental pollution drag effect (%)	−0.1143	0.0042	0.0082	−0.0387	0.2501
Total drag effect (%)	−0.8347	0.0137	0.0248	−0.1045	0.6891

**Table 6:** SDM estimation results and effect decomposition of the upper, middle, and lower reaches of the Yellow River Basin.

Area	Upper Yellow River				Middle Yellow River				Lower Yellow River			
Variable	SDM	Direct Effect	Indirect Effect	Total Effect	SDM	Direct Effect	Indirect Effect	Total Effect	SDM	Direct Effect	Indirect Effect	Total Effect
lnK	0.6826 ***	0.6496 ***	−0.3036 ***	0.3460 ***	0.8360 ***	0.8130 ***	−0.2210 **	0.5920 ***	−0.7548 ***	−0.7307 ***	0.3403 ***	−0.3904 ***
	(9.3300)	(9.5500)	(−2.6600)	(3.3500)	(13.5900)	(13.0900)	(−2.4200)	(5.3700)	(−7.5700)	(−7.5000)	(2.8700)	(−4.3300)
lnAL	−0.1540 *	−0.3160 ***	−1.3470 ***	−1.6630 ***	−0.0088	−0.0108	0.0016	−0.0092	−0.0108 *	−0.0116 **	−0.0095	−0.0211
	(−1.9500)	(−3.2400)	(−4.1400)	(−4.1600)	(−0.2200)	(−0.2500)	−0.0200	(−0.0700)	(−1.9300)	(−2.1400)	(−0.6800)	(−1.3700)
lnE	−0.0139 *	−0.0204 **	−0.0621 ***	−0.0825 ***	0.0080 *	0.0157 ***	0.0642 ***	0.0799 ***	−0.0007	−0.0017	−0.0182 ***	−0.0199 ***
	(−1.7800)	(−2.3600)	(−2.5600)	(−2.7700)	−1.9500	−3.4400	−3.5700	−3.8700	(−0.6900)	(−1.5900)	(−4.9000)	(−4.6100)
lnW	0.0087	0.0161	0.0695	0.0856	0.0435**	0.0447**	0.0117	0.0564	0.0241 ***	0.0243 ***	0.0050	0.0294
	(0.2500)	(0.4100)	(0.5900)	(0.6000)	(2.3700)	(2.0200)	(0.2000)	(0.7400)	(4.9100)	(4.9700)	(0.3800)	(1.9200)
lnR	0.1280 ***	0.1770 ***	0.4140 **	0.5910 **	0.06810 **	0.08290 **	0.1180	0.2010 *	0.0115 *	0.0129 *	0.0218	0.0347
	(2.6100)	(2.9600)	(2.1900)	(2.5300)	(2.3500)	(2.4400)	(1.3700)	(1.7800)	(1.6600)	(1.7800)	(1.0900)	(1.4200)
lnB	−0.0518 *	−0.0687 **	−0.1570 *	−0.2250 **	0.0224	0.0323	0.0875	0.1200	0.0158 ***	0.0133 ***	−0.0424 ***	−0.0291 **
	(−1.7900)	(−2.0900)	(−1.9400)	(−2.2000)	−1.3100	−1.4800	−1.0700	−1.2200	−4.0000	−3.3000	(−3.7600)	(−2.2200)
lnS	0.0417 *	0.0291	−0.1070	−0.0783	−0.0363 ***	−0.0550 ***	−0.160 ***	−0.215 ***	0.0014	0.0022	0.0127	0.0148
	(1.8400)	(0.9400)	(−1.3600)	(−0.7400)	(−3.5200)	(−4.1600)	(−3.2300)	(−3.6700)	(0.3800)	(0.5200)	(1.2300)	(1.2100)
lnD	−0.0147	−0.0044	0.0906	0.0862	0.0211	0.0300 **	0.0752 ***	0.1050 ***	−0.0105 ***	−0.0110 ***	−0.0063	−0.0172 **
	(−0.7500)	(−0.2100)	(1.4300)	(1.1200)	(1.5700)	(2.2900)	(2.8800)	(3.3800)	(−4.0600)	(−4.5300)	(−0.8400)	(−2.0800)
WlnK	−50.4400 ***	—	—	—	−0.6270 ***	—	—	—	4.7140 ***	—	—	—
	(−5.2200)	—	—	—	(−10.7500)	—	—	—	−4.0800	—	—	—
WlnAL	−0.7220 ***	—	—	—	0.0103	—	—	—	−0.0046	—	—	—
	(−4.1000)	—	—	—	−0.2500	—	—	—	(−0.4300)	—	—	—
WlnE	−0.0304 **	—	—	—	0.0200 **	—	—	—	−0.0134 ***	—	—	—
	(−2.0400)	—	—	—	−2.3800	—	—	—	(−4.9600)	—	—	—
WlnW	0.0337	—	—	—	−0.0207	—	—	—	−0.0035	—	—	—
	−0.5200	—	—	—	(−0.9900)	—	—	—	(−0.3700)	—	—	—
WlnR	0.1880 *	—	—	—	0.0006	—	—	—	0.0133	—	—	—
	−1.8400	—	—	—	−0.0200	—	—	—	−0.9100	—	—	—
WlnB	−0.0712	—	—	—	0.0204	—	—	—	−0.0371 ***	—	—	—
	(−1.5800)	—	—	—	−0.7100	—	—	—	(−4.5100)	—	—	—
WlnS	−0.0814 **	—	—	—	−0.0394 **	—	—	—	0.0098	—	—	—
	(−2.0200)	—	—	—	(−2.1000)	—	—	—	−1.2900	—	—	—
WlnD	0.0596	—	—	—	0.0149	—	—	—	−0.0021	—	—	—
	−1.6300	—	—	—	−1.1000	—	—	—	(−0.3900)	—	—	—

Note: ***, **, * means passing the significance test at the levels of 1%, 5%, and 10%, respectively. The values in () are statistical values, the same as below.

**Table 7:** Results of the economic growth drag effects of the upper, middle, and lower reaches of the Yellow River Basin.

Area	Upper Yellow River				Middle Yellow River				Lower Yellow River			
Variable	SDM	Direct Effect	Indirect Effect	Total Effect	SDM	Direct Effect	Indirect Effect	Total Effect	SDM	Direct Effect	Indirect Effect	Total Effect
Capital stock elasticity coefficient (α)	0.6826	0.6496	−0.3036	0.3460	0.8360	0.8130	−0.2210	0.5920	−0.7548	−0.7307	0.3403	−0.3904
Energy resource elasticity coefficient (β)	−0.0139	−0.0204	−0.0621	−0.0825	0.0080	0.0157	0.0642	0.0799	−0.0007	−0.0017	−0.0182	−0.0199
Water resource coefficient (φ)	0.0087	0.0161	0.0695	0.0856	0.0435	0.0447	0.0117	0.0564	0.0241	0.0243	0.0050	0.0294
Elasticity coefficient of land resources (ν)	0.1280	0.1770	0.4140	0.5910	0.0681	0.0829	0.1180	0.2010	0.0115	0.0129	0.0218	0.0347
Industrial wastewater discharge elasticity coefficient (δ)	−0.0518	−0.0687	−0.1570	−0.2250	0.0224	0.0323	0.0875	0.1200	0.0158	0.0133	−0.0424	−0.0291
Industrial SO_2_ emission elasticity coefficient (ω)	0.0417	0.0291	−0.1070	−0.0783	−0.0363	−0.0550	−0.1600	−0.2150	0.0014	0.0022	0.0127	0.0148
Industrial smoke emission elasticity coefficient (ψ)	−0.0147	−0.0044	0.0906	0.0862	0.0211	0.0300	0.0752	0.1050	−0.0105	−0.0110	−0.0063	−0.0172
Average annual growth rate of labor (n)	0.0079	0.0079	0.0079	0.0079	0.0070	0.0070	0.0070	0.0070	0.0047	0.0047	0.0047	0.0047
Natural resource drag effect (%)	0.0009	0.0013	−0.0060	0.0081	0.3216	−0.3527	−0.0876	−0.4241	−0.0018	0.0019	−0.0019	−0.0023
Environmental pollution drag effect (%)	0.0005	0.0008	−0.0063	0.0074	0.1100	0.1415	0.0603	0.2455	−0.0014	−0.0016	0.0124	−0.0088
Total drag effect (%)	0.0014	0.0021	−0.0123	0.0156	0.2116	−0.2111	−0.0274	−0.1786	−0.0032	−0.0035	0.0105	−0.0111

## Data Availability

Not applicable.

## Abbreviations

The following abbreviations are used in this manuscript:

CGE	Computable general equilibrium
SDM	Spatial Durbin model
GDP	Gross domestic product
SAR	Spatial autoregressive model
LLC	Levin, Lin, and Chu test
SEM	Spatial error model
IPS	Im, Pesaran, and Shin test
LR	Likelihood ratio test
